# Inflammatory Mediation of Heat Stress-Induced Growth Deficits in Livestock and Its Potential Role as a Target for Nutritional Interventions: A Review

**DOI:** 10.3390/ani11123539

**Published:** 2021-12-13

**Authors:** Micah S. Most, Dustin T. Yates

**Affiliations:** Department of Animal Science, University of Nebraska-Lincoln, Lincoln, NE 68583, USA; micah.most@huskers.unl.edu

**Keywords:** climate change, cytokines, feedlot, hyperthermia, inflammation, muscle, nutraceuticals, TNFα

## Abstract

**Simple Summary:**

Heat stress is a persistent challenge for livestock producers. Molecular changes throughout the body that result from sustained heat stress slow muscle growth and thus are detrimental to carcass yield and value. Feedlot animals are at particularly high risk for heat stress because their confinement limits their ability to pursue shade and other natural cooling behaviors. Changes in infrastructure to reduce the impact of heat stress are often cost-prohibitive, but recent studies have revealed that anti-inflammatory therapies may help to improve growth deficits in heat-stressed animals. This review describes the conditions that cause heat stress and explains the role of inflammation in muscle growth impairment. Additionally, it discusses the potential for several natural anti-inflammatory dietary additives to improve muscle growth outcomes in heat-stressed livestock.

**Abstract:**

Heat stress is detrimental to well-being and growth performance in livestock, and systemic inflammation arising during chronic heat stress contributes to these poor outcomes. Sustained exposure of muscle and other tissues to inflammation can impair the cellular processes that facilitate muscle growth and intramuscular fat deposition, thus reducing carcass quality and yield. Climate change is expected to produce more frequent extreme heat events, increasing the potential impact of heat stress on sustainable livestock production. Feedlot animals are at particularly high risk for heat stress, as confinement limits their ability to seek cooling from the shade, water, or breeze. Economically practical options to circumvent heat stress in feedlot animals are limited, but understanding the mechanistic role of inflammation in heat stress outcomes may provide the basis for treatment strategies to improve well-being and performance. Feedlot animals receive formulated diets daily, which provides an opportunity to administer oral nutraceuticals and other bioactive products to mitigate heat stress-induced inflammation. In this review, we examine the complex associations between heat stress, systemic inflammation, and dysregulated muscle growth in meat animals. We also present evidence for potential nutraceutical and dietary moderators of inflammation and how they might improve the unique pathophysiology of heat stress.

## 1. Introduction

Heat stress is a long-standing barrier to global livestock production due to its impact on animal health and performance [1,2]. Indeed, the extended periods of heat stress common to much of the inhabited world increase morbidity and mortality rates, reduce growth and efficiency and diminish the amount and quality of meat or milk produced by each animal [3,4]. Moreover, the continued emergence of climate change makes mitigation strategies for the effects of heat stress increasingly important to the sustainability of the livestock industry [5]. In the US and Europe, heat waves are projected to occur with greater frequency, intensity, and duration over the next century, including in regions for which such events are historically uncommon [6]. Greater peak daytime temperatures during these events are detrimental for livestock [7], but elevated nighttime temperatures substantially worsen outcomes by reducing windows for heat dissipation [8]. Animal well-being experts have noted that livestock housed in confined areas such as feedlots and dairies are particularly susceptible to heat stress, as they are often restricted in their ability to engage in alleviation behaviors such as seeking shade or wading in water when these options are not provided by the infrastructure of the facility [9,10,11]. Although confinement is necessary during certain stages of food animal production, greater mortality and morbidity from heat stress in these populations is a major animal welfare issue. Greater death loss and reduced production associated with heat stress also threaten the economic sustainability of the livestock industry. For example, estimates based on 2018 beef prices indicate that each feedlot steer lost during a heat event costs the producer about $5000 [12]. Most animals survive even severe heat events, however, and heat stress-associated reductions in growth and productivity cost the industry five to ten-fold more annually than heat stress-associated death loss [12]. For beef cattle, heat stress during the finishing phase reduces carcass yield and quality traits such as marbling and tenderness, resulting in billions of dollars in lost revenue each year [3,13,14]. Unfortunately, strategies to improve health and production outcomes in heat-stressed animals are limited by a poor understanding of the physiological mechanisms that dictate these responses. However, recent research has indicated that chronic heat stress induces systemic inflammatory responses, which appears to be one mechanism facilitating heat stress pathologies. This review highlights the evidence for how chronic heat stress induces systemic inflammation and its potential mediating role in poor growth and body composition outcomes, as well as how it might be a target for intervention strategies.

## 2. The Impact of Heat Stress in Livestock

### 2.1. Heat Stress Conditions

**Figure 1:**
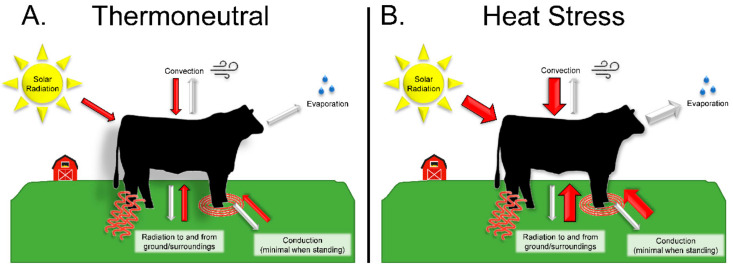
Thermal energy is exchanged between the animal and environment by 3 main processes: convection, conduction, and radiation. In livestock, conduction is generally limited to recumbent positions. Evaporation of sweat and moisture from the respiratory tract is an important mechanism for heat dissipation. Under thermoneutral conditions (**A**), thermal input from the environment is roughly equal to dissipation, and the animal does not expend additional energy to maintain homeostatic body temperature. During heat stress (**B**), thermal input exceeds normal dissipation, and the animal must engage in additional processes for heat dissipation to maintain a stable body temperature. When heat stress is extreme, this homeostasis may be lost, resulting in hyperthermia.

Mammalian animals become heat stressed when heat is produced and absorbed by their body at a greater rate than it is dissipated, resulting in hyperthermia [11]. The mechanisms for body heat dissipation (i.e., conduction, convection, and evaporation) are greatly influenced by environmental conditions [15], as illustrated in Figure 1.

Heat moves down its gradient, and thus ambient conditions that heat the ground and other surroundings to temperatures that exceed body temperatures markedly limit the effectiveness of heat dissipation by conduction. Dissipation via convection requires adequate air movement and falls substantially during periods of little to no wind, and high relative humidity in the ambient air limits evaporative heat dissipation associated with sweating and panting [15]. Moreover, clear skies that lack cloud cover result in greater heat input from solar radiation [16]. Consequently, livestock are at the greatest risk for heat stress on hot, clear days with high humidity and minimal wind speed [17]. Most agricultural regions of the world experience periods of seasonal heat waves, defined as three or more consecutive days of unusually hot daytime conditions with limited nighttime cooling [6]. Of course, heat waves are not always dependent upon season, and heat stress can occur any time animals face thermal loads to which they are unacclimated [8], which can be dictated by more than just ambient air temperatures. After studying the effects of humidity on milk production for years, Hudson Kibler popularized the use of the Temperature-Humidity Index (THI) in the mid-1960s to estimate thermal loads in dairy cows under various air temperature/humidity combinations [18]. The use of this index, which was based on Earl Thom’s model for describing human discomfort [19,20], has been expanded to other livestock species in the decades since, and in 1970 THI values were used to create the risk categories of the Livestock Weather Safety Index [21]. For beef cattle in feedlots, THI values of less than 74 are classified as *Normal* and would not be expected to impose stress [22]. Conversely, THI values above 75 place feedlot cattle in the increasingly concerning stress categories of *Alert* (75 to 78), *Danger* (79 to 84), and *Emergency* (greater than 84) [22], which would be expected to result in the responses and outcomes summarized in Table 1.

Most sheep breeds are more heat tolerant than cattle and do not typically exhibit signs of stress at THI values of less than 82 [24]. Moderate heat stress responses would be expected from sheep at THI values of 82 to 84, more severe responses would be expected at 84 to 86, and the most extreme outcomes (including substantial increases in mortality rates) would only be expected when THI values exceed 86 [24]. Although THI values provide more accurate estimates of thermal load in livestock than air temperatures alone, they do not account for the contribution of solar radiation and wind speeds [26,27]. In 1981, however, dairy researchers in Florida expanded the THI algorithm to include both net radiation and air movement in addition to temperature and humidity, resulting in the Black Globe-Humidity Index (BGHI) [28]. Named for the solar radiation-absorbing, black-painted globe placed around the thermometer, BGHI more accurately predicts heat stress indicators such as changes in rectal temperatures and respiratory rates than THI alone. However, because BGHI requires additional equipment and measurements and only differs from THI under specific conditions, THI is more commonly used for livestock.

### 2.2. Consequences of Heat Stress

#### 2.2.1. Hyperthermia

Perhaps the greatest mediator of physiological changes in heat-stressed animals is the elevation of body temperature. In feedlot sheep, rectal temperatures were increased by 0.5 °C to 1.5 °C within hours of initiating heat stress. Moreover, rectal, corneal, and skin temperatures remained elevated until heat stress ceased weeks later, even when substantial overnight cooling occurred [29,30,31]. For comparison, the magnitude of these increases were comparable to the febrile response observed when sheep were injected with bacterial endotoxin [32,33]. In cattle and sheep, extreme heat stress-induced hyperthermia can cause fatal damage to the brain and other vital organs [34,35]. In more moderate cases, hyperthermia decreases appetite and impairs molecular mechanisms for growth and metabolic efficiency [29,30,31]. 

#### 2.2.2. Hyperventilation

Increasing respiratory rate, or panting, is an important mechanism by which heat-stressed livestock can dissipate body heat [24]. In fact, greater efficiency of panting appears to be a factor in the enhanced thermotolerance of some cattle breeds [36]. Respiratory rate is a frequently used indicator of heat stress in feedlot cattle because it is an objective measurement, is influenced predictably, and does not tend to lag behind changes in ambient conditions [26]. Cattle generally exhibit respiratory rates of about 60 breaths/minute in the absence of stress but can more than double this rate during even moderate heat stress [7]. Although an effective cooling mechanism, elevated respiratory rate can have negative impacts on blood chemistry when sustained. Hyperventilation for as little as 2 h can decrease blood CO_2_, which increases blood pH and results in respiratory alkalosis [37,38]. 

#### 2.2.3. Endocrine Changes

Predictably, heat stress increases the secretion and activity of several stress hormones. Circulating concentrations of adrenaline, noradrenaline, cortisol, prolactin, vasopressin, and cytokines have all been shown to be elevated in response to chronic heat stress [29,31,38,39,40]. Moreover, tissue sensitivity to stress hormones can be increased following sustained periods of heat stress [41,42]. The increased tone of stress hormones in turn affects a broad range of metabolic hormones. Decreased circulating concentrations of the thyroid hormones thyroxine (T4) and triiodothyronine (T3) have been observed in heat-stressed cattle and small ruminants [38,43], which is in fact believed to aid in thermal acclimatization [44]. However, thyroid hormones also potentiate the anabolic outcomes of growth hormone (GH) signaling [45,46], an effect that is lost with the diminished secretion of the former. Moreover, circulating GH concentrations are reduced in heat-stressed cattle [47], whereas concentrations of the appetite-suppressing hormone leptin are increased due to greater stimulation from cortisol [25,48]. 

#### 2.2.4. Anorexia and Poor Growth Performance

Among the greatest barriers to feedlot production in chronically heat-stressed livestock is reduced feed intake [49,50]. Indeed, high THI has a strong inverse correlation with dry matter intake [51], which predictably reduces the rate and efficiency of weight gain and if sustained can ultimately affect the animal’s health and well-being [9,52,53]. A recent study of heat-stressed feedlot wethers found that they had less fat-free lean tissue relative to their total bodyweight [54]. Moreover, loin muscles and 9th–12th rib cutouts from these heat-stressed wethers contained less fat [31]. Similar observations were reported in goats, as heat stress decreased loin-eye area and fat score [25]. In cattle, heat stress disproportionally reduced subcutaneous fat deposition relative to other fat deposits, perhaps to diminish its insulating effect on heat dissipation [55]. 

Although the mediating role of reduced intake is well documented, results from pair-feeding studies (i.e., studies in which the amount of feed offered to thermoneutral control animals is adjusted to be equivalent to the amount consumed by heat-stressed animals) have demonstrated that heat stress reduces growth performance through other mechanisms as well. These intake-independent changes may vary with the intensity and duration of the heat stress but are typically associated with disruptions in metabolic processes [56,57,58]. Large-scale pair-feeding studies in feedlot cattle are rare due to their high costs and logistical difficulties, but a study performed in pair-fed mice estimated that around 50% of heat stress effects were due to intake-independent factors [59]. Such mechanisms alter endocrine and metabolic aspects of growth regulation, which helps to explain poor growth performance observed in heat-stressed feedlot lambs, even when thermoneutral counterparts were pair-fed [31,54]. One prominent example of an intake-independent mechanism would be cellular oxidative stress, as greater production of free radicals increases plasma membrane and mitochondrial damage, impairs metabolic signaling, increases protein catabolism, and decreases protein synthesis [60]. In swine, as little as 12 h of heat stress was enough to induce oxidative stress in muscle, leading to disruption in multiple regulatory processes [61]. These and other intake-independent changes in tissue growth during heat stress are associated with altered gene expression. Indeed, the expression for stress response mediators such as c-Fos, acute-phase proteins, RNA binding proteins, adrenergic signaling components, and protein ubiquitination was greater in muscle and fat tissues from heat-stressed lambs [41,42]. However, not all changes in gene expression are negative. For example, muscle from chronically heat-stressed cattle and goats expressed greater mRNA for heat shock proteins [25,62], which are key facilitators of protein stability that protect against heat-induced protein damage [63]. In fact, single nucleotide polymorphisms for heat shock protein 70 identified in two African cattle breeds appear to contribute to their enhanced thermotolerance [64].

### 2.3. Common Abatement Strategies for Heat Stress

Because feedlot animals are almost always housed outdoors, they are fully subject to extremes in ambient temperatures, humidity, and solar radiation conditions. For these animals, the risk of heat stress is increased not only by their limited ability to pursue natural shade or breeze but also by the greater metabolic heat load produced by their high-energy diets [65]. For decades, heat stress abatement strategies in confined livestock have centered on three main methods for mitigating high thermal load: (1) protection of animals via environmental modifications, (2) genetic selection for greater heat tolerance, and (3) altering nutritional management approaches to best fit the changing nutritional requirements [66].

#### 2.3.1. Protection via Environmental Modification

A number of different types of physical barriers have been used to reduce thermal input by blocking radiation and to increase heat dissipation by enhancing convection and evaporation. Structures built to provide feedlot cattle with greater shade have been associated with reduced respiration rates and core body temperatures [26,40,49], which is consistent with a less severe stress response. Providing shade to feedlot cattle also increased their dry matter intake, average daily gain, and final body weight for the finishing period, and increased the percentage of animals grading as USDA Choice at harvest [67]. The efficacy of shade structures are of course dependent upon their design and the materials used, which must be weighed against the cost [9,68]. The use of sprinklers to wet the ground in pens can provide brief reductions in radiation from the soil surface but can also increase air humidity and may create mud buildup [69]. Using sprinklers to wet the cattle directly can reduce the thermal load in drier climates but is ineffective or even harmful in areas with high humidity, as droplet barriers in the hair coat can disrupt non-evaporative heat dissipation [70,71]. Moreover, cattle responses to heat stress are worsened upon discontinuation of sprinklers after prolonged use due to de-acclimation [12]. Thus, sprinkler cooling is best suited for short-term use in low-humidity environments. A recent review by Dahl et al. [72] provides more detail about the effectiveness and limitations of environmental modifiers on heat stress abatement.

#### 2.3.2. Genetic Selection for Heat Tolerance

Genetic selection for traits that reduce the impact of thermal load is an effective strategy for combating heat stress, particularly in areas for which heat stress is common. Although a popular selection criterion in feedlot cattle for most of the last century has been darker coat color, these absorb substantially more solar radiation than lighter coats [65,73]. Consequently, cattle with dark coats had elevated body temperatures and panting rates during mild to moderate heat stress and greater mortality rates from extreme heat events compared to those with lighter coats [35,74]. Similarly, hair sheep with brown or black coats exhibited greater rectal temperatures and respiratory rates than sheep with white coats due to their increased absorption of solar radiation [75]. Thermal tolerance can also be improved by selecting animals with greater *Bos indicus* influence, as these breeds are more adapted to hot and humid environments because of their geographical origin [76]. Regardless of coat color, *Bos indicus* breeds have lower metabolic rates than *Bos taurus* breeds and thus produce less metabolic heat [77,78]. They also express a greater capacity for sweating and non-evaporative cooling [36,76,79]. As a result, cattle with complete or partial *Bos indicus* composition exhibit less severe hyperthermia, hyperventilation, anorexia, and growth restriction when experiencing heat stress [80,81]. Additionally, there is substantial variation in thermal tolerance among *Bos taurus* breeds. For example, Senepol and Romosinuano cattle presented less severe increases in body temperature and respiratory rates than Angus cattle during severe heat stress [81]. Additionally, Mertolenga cattle were more successful in moderating hyperthermic responses to heat stress than Alentejana, Frisian, or Limousine cattle, as they appeared to utilize a more robust combination of heat dissipation mechanisms [82]. Of course, selecting for improved tolerance to heat stress must be weighed against the potential for decreased growth efficiency, carcass yield, and quality traits associated with some heat-tolerant breeds, and these decisions must be made in advance of heat events [83]. 

#### 2.3.3. Nutritional Management of Heat-Stressed Livestock

Digestion and metabolism are heat-generating processes, and thus reduced intake is actually a coping mechanism to limit the body’s endogenous heat increment [12]. This creates two potential objectives for feeding strategies during heat stress conditions: (a) helping to reduce the animal’s digestive/metabolic heat increment and (b) minimizing the impact of lower intake on growth performance. The amount of heat produced by digestion/metabolism is dependent upon the diet’s components. Specifically, fibrous roughages are associated with large heat increments, whereas nutrient-dense concentrate ingredients are associated with more moderate heat increments [12]. Rations used by feedlots typically contain mixtures of roughages and concentrate ingredients, and thus heat increment of the total diet can be manipulated by reducing the amount of feed consumed (heat-stressed animals often do this voluntarily) or by altering the roughage/concentrate ratios of the ration [17]. Indeed, temporarily restricting dry matter intake to approximately 75% of normal *ad libitum* intake was shown to reduce body temperatures in feedlot cattle, even when diets contained a relatively high proportion of roughage [74,84]. In addition to changing ration formulations or reducing the amounts fed, a number of different supplements offer varying degrees of protection from heat stress. In feedlot animals, nutrient supplements as diverse as electrolytes, yeast, rumen-protected carbohydrates, and free ferulic acid created improvements ranging from greater water intake to reduced body temperatures, respiration rates, blood cortisol concentrations, and protein oxidation rates [23,53,85,86]. Dietary additives such as the β agonists—ractopamine and zilpaterol—were also effective in moderating heat-induced hyperthermia and hyperventilation [29,31,87]. Despite variability in the benefits they provide to heat-stressed animals, supplements are attractive options because they do not require the financial investments of structural interventions and are not associated with the potential lost production of thermotolerant genetic selection.

## 3. The Role of Inflammation in Heat Stress

**Figure 2:**
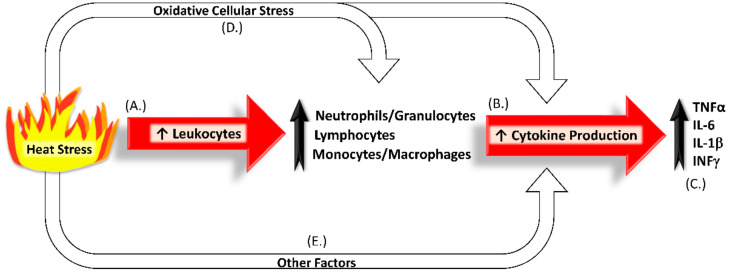
Heat stress increases components of systemic inflammation, including circulating populations of total leukocytes, granulocytes, lymphocytes, and monocytes (**A**) [29,30,31,40,67]. Along with other tissues, these circulating leukocytes produce inflammatory cytokines in greater amounts (**B**) [91,92,93], resulting in greater cytokine concentrations in the bloodstream (**C**) that contribute to the enhanced inflammatory tone observed in heat-stressed livestock [31,94,95,96]. At the same time, oxidative cellular stress (**D**) and other factors not discussed in this review (**E**) can further stimulate cytokine synthesis and release [60,97,98].

The impact of heat stress on the immune system is complex and dynamic. When heat stress is associated with a spike in circulating cortisol due to reduced nutrient intake, the immune system as a whole can be suppressed [88]. However, recent studies show that heat stress often results in little or no increases in circulating cortisol, particularly when there are only modest reductions in dietary intake [31,40,89]. Consequently, heat stress frequently increases inflammatory components of the immune system [90], as summarized in Figure 2. 

Several mechanisms described in the literature appear to contribute to the link between heat stress and greater inflammatory tone. In rats, for example, four weeks of daily heat stress increased activation of leukocytes in the spleen [99], which would help to explain their greater concentrations in the bloodstream of chronically heat-stressed livestock. Indeed, several studies report changes in circulating leukocyte populations in heat-stressed feedlot animals, as commercial finishing heifers without access to shade during mild to moderate heat stress exhibited greater neutrophil and lymphocyte concentrations as well as neutrophil-to-lymphocyte ratios compared to those with shade [40,67]. Feedlot lambs that were exposed to substantial daytime heat stress for 3 to 4 weeks exhibited greater circulating concentrations of total leukocytes, lymphocytes, monocytes, and granulocytes regardless of whether controls were or were not pair-fed [29,31]. Inflammatory cytokines are produced by these leukocytes [91,92,93] as well as by muscle and other tissues [99,100,101,102]. In fact, as little as 12 h of heat stress in pigs was sufficient to increase muscle content of the inflammation-mediating kinase IKKα and, in turn, the phosphorylation of its nuclear target NFκB, which predictably increased gene expression for TNFα and other inflammatory cytokines [102]. A study performed in immortalized C2C12 skeletal muscle cells showed that increased expression for inflammatory cytokines may also result from greater assembly and activation of the inflammation-mediating NLRP3 inflammasome, as gene expression for all necessary components of this oligomer are increased after a 15-h period of in vitro heat stress [103]. Moreover, studies in rodents indicate that the heat shock protein HSP70 plays a key upstream role in mediating heat stress induction of inflammatory pathways [99,100,101]. For example, in muscle and other tissues from heat-stressed rats and mice, enhanced HSP70 activation of the toll-like receptor TLR4 was associated with the increased nuclear activity of NFκB and AP1, which are central transcription factors for myriad inflammatory cytokines. Likewise, 7-day heat stress in sheep increased HSP70, NFκB, and TNFα in the loin muscle [104]. Oxidative cellular stress is also a major stimulant of cytokine secretion [97], and thus the increased production of reactive oxygen species during heat stress may play a large role in the greater inflammatory tone observed in chronically heat-stressed livestock [60,98]. Indeed, muscle from heat-stressed lambs showed evidence that greater production of reactive oxygen species further increased NFκB-mediated cytokine transcription by stimulating its effecter, MAP Kinase [104]. The result of these combined mechanisms is greater systemic inflammation characterized in part by elevated circulating concentrations of inflammatory cytokines. In a recent study, feedlot lambs exhibited greater blood concentrations of TNFα by the 2nd week of heat stress [31]. Likewise, the response of dairy cattle to heat stress included increased circulating concentrations of leukocyte-derived TNFα, IL-6, IL1β, and IFNγ, despite the concurrent presence of modest increases in circulating cortisol [94,95,96]. 

### 3.1. Inflammatory Regulation of Muscle Growth

Postnatal skeletal muscle growth occurs via muscle fiber hypertrophy, which is facilitated by quiescent progenitors called satellite cells that are stored in the basal lamina of fibers during prenatal development [105,106]. In response to growth promoters, satellite cells are activated into myoblasts, which proliferate through several cycles of replication and then undergo terminal differentiation before fusing with existing muscle fibers [107]. This accumulation of additional myonuclei increases the DNA content of the muscle fiber and, in turn, its capacity for protein synthesis [108,109,110]. 

**Figure 3:**
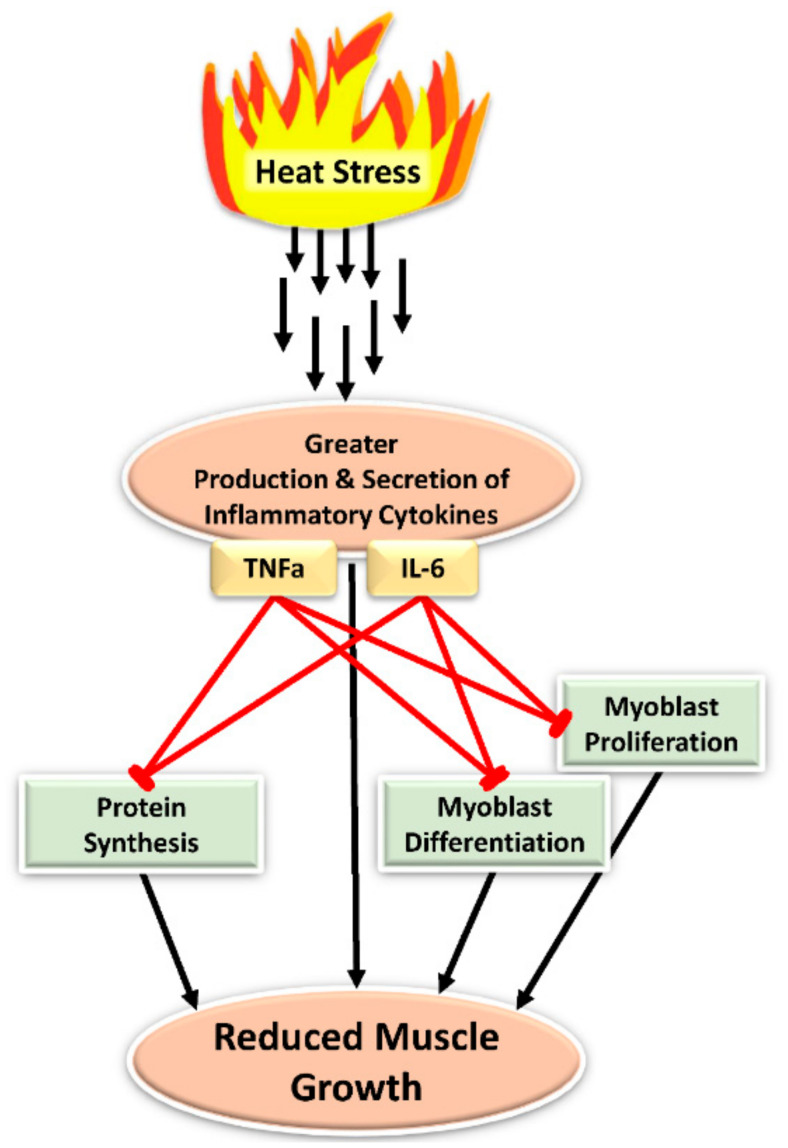
Heat stress-induced inflammatory factors interfere with the major processes that facilitate muscle growth. Specifically, heat stress increases circulating leukocyte populations [29,30,31,40,67], which in turn contribute to greater circulating inflammatory cytokines, including TNFα and IL-6 among others [91,92,93]. Exposure of myoblasts (i.e., muscle stem cells) to TNFα or IL-6 can reduce their proliferation and differentiation rates, which is detrimental to muscle growth [111,112]. Additionally, exposure of skeletal muscle itself to TNFα or IL-6 reduces its protein synthesis [119,120,121] in part by re-appropriating amino acid utilization [122].

Myoblast function, protein synthesis, and muscle growth are influenced substantially by cytokines, as illustrated in Figure 3. When myoblasts isolated from fetal sheep were incubated with TNFα or IL-6, they exhibited reductions in proliferation and differentiation rates [111]. In cells from adults, however, inflammatory cytokines primarily disrupt myoblast differentiation and fusion processes and may in fact even increase proliferation rates [112]. Several signaling mechanisms appear to contribute to cytokine-induced impairment of myogenic commitment and differentiation in myoblasts. Primary skeletal muscle fibers and myoblasts isolated from mice expressed less of the transcription factor MyoD when incubated with TNFα [113]. Because MyoD is critical in transitioning myoblasts out of the replication phase and into differentiation and fusion, these TNFα-spiked incubations ultimately contained greater numbers of myoblasts but smaller-diameter fibers [113]. Incubation of myoblasts with TNFα and IL-6 also reduced their expression of the terminal differentiation factor myogenin [114]. Several studies indicate that the anti-differentiation effects of TNFα, IL-6, TWEAK, and other inflammatory cytokines are mediated by the downstream actions of TNF receptor-associated factor 6 (TRAF6), which not only activates canonical NFκB pathways but also JNK, p38 MAPK, and AMPK pathways [115,116]. Indeed, these later pathways help to explain NFκB-independent disruption of the differentiation process that limits muscle growth [116,117,118].

TNFα also suppressed protein synthesis in human primary myoblasts co-incubated with IGF-1 [119]. Because myoblasts play a rate-limiting role in muscle growth, their functional impairment by cytokines causes a predictable reduction in muscle mass. Early studies showed that exogenous administration of TNFα or IL-1α to rats induced weight loss and diminished their whole-body protein content [120,121]. Elevated concentrations of TNFα and IL-6 in circulation promote repartitioning of amino acids away from protein synthesis in skeletal muscle [122]. In fact, heightened systemic inflammation is believed to be the primary cause for reduced performance in feedlot cattle with respiratory infections, as these animals exhibit decreased average daily gain, carcass weight, fat thickness, and marbling [123,124]. Even prenatal exposure to inflammatory cytokines can restrict muscle growth, as maternofetal inflammation in rats decreased fetal mass, hindlimb cross-sectional area, and muscle fiber size [125]. The effects of inflammation on myoblast function and protein synthesis in muscle coincide with disrupted insulin signaling. Indeed, primary rat soleus muscle incubated with TNFα or IL-6 exhibited substantially reduced rates of insulin-stimulated Akt phosphorylation [126].

### 3.2. Methods for Targeting Inflammation during Heat Stress

Systemic inflammation presents a potential target for therapeutic strategies to improve well-being and growth outcomes in heat-stressed livestock. Moreover, feedlot animals are fed mixed rations daily, which provides an opportunistic avenue to administer oral supplements that target this heightened inflammation and the oxidants that often mediate it. In this section, we discuss several products that have potential as dietary interventions for inflammation and oxidative stress in heat-stressed animals. These are also summarized in Table 2. 

#### 3.2.1. Brown Seaweed & Seaweed Extract

Brown seaweed species such as *Sargassum latifolium* and *Ascophyllum nodosum* are used frequently as supplements due to their potent antioxidant and anti-inflammatory properties [127]. Indeed, brown seaweed extract was found to be effective in reducing oxidative stress and inflammation associated with residual fescue toxicity in feedlot cattle, which helped to recover carcass merit in these animals [128,129,130]. When included at up to 4% of the ration for feedlot sheep, dried whole brown seaweed moderated heat stress-induced increases in circulating leukocytes, TNFα, and IL-6 in a dose-dependent manner [131]. This in turn reduced hyperthermia and hyperventilation and recovered growth in these heat-stressed sheep. Similar effects on body temperature were observed in heat-stressed Boer goats supplemented daily with brown seaweed extract [132].

#### 3.2.2. Resveratrol

Resveratrol is a bioactive extract found in grapes and other vegetation that has documented antioxidant and anti-inflammatory properties [133]. Although information regarding its effects in heat-stressed ruminants is limited, it has been studied in rodents and poultry. When orally supplemented to Sprague-Dawley rats during an intense 3-day heat stress period, resveratrol suppressed the rise in hepatic TNFα production and in gene expression for other cytokines and inflammatory factors [134]. Although this study did not assess systemic inflammation or growth, it is reasonable to speculate that improvements in these outcomes were possible for resveratrol-supplemented heat-stressed rats. Oral resveratrol supplementation also reduced tissue inflammation in heat-stressed chickens [135]. Of course, the differences in digestive physiology of ruminants compared to non-ruminants and poultry may affect the bioavailability of orally supplemented resveratrol, and thus investigation in ruminant livestock proper is warranted.

#### 3.2.3. Turmeric Curcumin

Curcumin is the bioactive compound found in the extract of roots from the *Curcuma longa* plant (commonly known as turmeric) [136]. It has a historic presence in Eastern medicine, and its antioxidant and anti-inflammatory properties have been demonstrated in sheep [137], among other animals. Like many supplements, studies using curcumin as an intervention for heat stress have not been performed in ruminant livestock to our knowledge. However, heat-stressed rats had less severe increases in renal TNFα, IL-6, and IL-1β expression when receiving oral supplementation of curcumin [138]. This was perhaps indicative of reduced inflammation in a broader range of tissues, as it also coincided with less muscle damage. Despite its well-documented anti-inflammatory properties, curcumin exhibits notably poor gastrointestinal absorption when administered orally [139]. Although strategies to improve bioavailability by identifying protective molecular structures and curcumin metabolism inhibitors are ongoing [140], it should be noted that this is currently a major limitation of oral curcumin supplementation. 

#### 3.2.4. Vitamin E + Selenium

The antioxidant activities of vitamin E and selenium (Se) when supplemented together and at supranutritional quantities have been well-characterized [141,142]. Additionally, administering oral vitamin E + Se to ewes prevented heat stress-induced increases in skeletal muscle gene expression for TNFα and NF-κB, which is consistent with reduced inflammation [104]. This in turn resulted in less severe hyperthermia, hyperventilation, and tachycardia under heat-stress conditions [104,143]. A similar study in goats also found that vitamin E + Se supplementation suppressed the heat stress-induced rise in body temperatures and respiratory rates [38]. These goats had less severe increases in circulating cortisol and prolactin and less severe reductions in circulating thyroid hormones. Although the free radical-scavenging activity of vitamin E + Se clearly improves the inflammatory status of heat-stressed livestock, it is worth noting that the high cost of these supplements can be limiting [144]. 

#### 3.2.5. ω-3. Polyunsaturated Fatty Acids

The capacity for ω-3 polyunsaturated fatty acids (ω-3 PUFA; e.g., eicosapentaenoic acid, EPA; docosahexaenoic acid, DHA) to mitigate inflammatory cytokine production and signaling has been documented in animals and cell lines [145,146,147,148,149,150,151,152]. Additionally, ω-3 PUFA can also increase the production of some anti-inflammatory cytokines [153,154]. Studies in cattle and sheep have shown that dietary supplementation of ω-3 PUFA can improve growth performance even in animals that are under little or no acute stress [155,156,157,158]. Thus, it is not surprising that daily supplementation of ω-3 PUFA-rich fish oil to heat-stressed feedlot lambs mitigated the rise in circulating granulocytes, granulocyte:lymphocyte ratios, body temperatures, and respiratory rates, which in turn improved muscle growth and rescued intramuscular fat content [159,160]. In heat-stressed dairy calves, fish oil reduced circulating TNFα and other inflammatory indicators, which was associated with reduced body temperatures and respiratory rates as well as improved feed efficiency [158]. Although fish oil is an effective and relatively inexpensive option for targeting inflammation, it should be noted that its taste and smell can create an aversion for some animals and can even result in reduced feed intake [161].

#### 3.2.6. Probiotics

There is substantial evidence for the protective effects of probiotics from yeast and other sources in heat-stressed poultry, but evidence in mammalian livestock species is less robust. However, a recent study in rats found that oral supplementation of probiotics from *L. acidophilus* and *S. cerevisiae* with and without selenium enrichment during a 42-day heat stress period moderated indicators of hepatic inflammation and oxidative stress [162]. Specifically, increased gene expression for TNFα, IL-6, COX-2, NFκB, HSP70, HSP90 was less severe in liver tissues from heat-stressed rats receiving the once-daily supplements. Additionally, heat stress-induced reductions in gene expression for the antioxidants GPX1, SOD1, and Nrf2 were less severe in probiotic-supplemented rats. A recent study in feedlot heifers found that dietary supplementation of yeast-based probiotics for 50 days prior to a 7-day heat-stress challenge reduced the rise in body temperature and respiration rates [85]. However, these were not concurrent with any changes in circulating leukocyte populations and may have been largely the result of increased water intake by supplemented heifers during the heat-stress period. In pigs that were heat-stressed for 28 days, the addition of live yeast to the diet modestly reduced circulating concentrations of TNFα compared to unsupplemented heat-stressed pigs [163]. However, TNFα concentrations of the heat-stressed pigs were comparable to respectively-supplemented, pair-fed thermoneutral controls. Moreover, yeast supplementation did not appear to improve heat stress-induced changes in body temperatures, respiratory rates, metabolic indicators, or growth efficiency. 

## 4. Conclusions

Chronic heat stress induces systemic inflammation characterized in part by greater circulating leukocyte and cytokine concentrations, which contribute to hyperthermia, hyperventilation, reduced growth performance, and compromised well-being. This greater inflammatory tone is particularly disruptive of muscle growth, as cytokines diminish the capacity of myoblasts to properly facilitate muscle fiber hypertrophy. In addition, inflammation reduces metabolic efficiency, as more nutrients are repartitioned for homeostatic mechanisms. Livestock maintained in confinement systems are at increased risk for heat stress due to their limited ability to self-protect by seeking shade or other cooler areas. However, well-being and productivity may be improved in these animals by dietary supplementation strategies that target the chronic inflammation associated with heat stress. Importantly, therapeutic reduction of systemic inflammation provides an opportunity to reduce the impact of heat stress in feedlot animals without manipulating their natural reduction in dietary intake, which is itself a heat stress-abating behavior. Moreover, limiting systemic inflammation and its impact on muscle growth processes in heat-stressed animals may also benefit their resumption of growth following the heat event. Heat stress is a major barrier to sustainable livestock production, and thus continuing the pursuit of nutraceutical strategies to improve health and productivity outcomes in heat-stressed food animals by targeting inflammation is warranted.

## Figures and Tables

**Table 1:** Temperature-Humidity Index (THI) ranges and associated responses in livestock.

THI Category	THI Ranges by Species		Respiration	Body Temperature Divergence (°C)	Behavioral Responses	Stress Level
	**Cattle ^1^**	**Sheep ^2^ & Goats ^3^**				
Normal	<74.0	<82.0	Normal	0.00	Normal	None
Alert	74.0 to 79.0	82.0 to 84.0	Steady but increased breathing	+0.11	Increased standing	Mild
Danger	79.1 to 84.0	84.1 to 86.0	Rapid, shallow breaths	+0.28	Bunching, ↓ feed intake	Moderate
Emergency	>84.0	>86.0	Deep, abdominal breaths with tongue extended	+0.56	Lingering at water source	Severe

^1^ [8,21,23]. ^2^ [15,24]. ^3^ [15,25]. ↓ = reduced.

**Table 2:** Potential Anti-inflammatory/Antioxidant Nutraceutical and Dietary Supplements for the Treatment of Heat Stress in Livestock.

Supplement	Source	Species Tested	References
Brown seaweed/extract	*Sargassum* & *Ascophyllum* spp.	Cattle, sheep, goats	[127,128,129,130,131,132]
Resveratrol	*Vitis* spp.	Rats, chickens	[133,134,135]
Turmeric curcumin	*Curcuma longa*	Sheep, rats	[136,137,138,139,140]
Vitamin E + Selenium	Commercial supplements	Sheep, goats,	[104,141,142,143,144]
ω-3 PUFA ^1^	EPA ^2^, DHA ^3^, fish oil	Cattle, sheep, mice, pigs, rats	[145,146,147,148,149,150,151,152,153,154,155,156,157,158,159,160,161]

^1^ Polyunsaturated fatty acid. ^2^ Eicosapentaenoic acid. ^3^ Docosahexaenoic acid.

## Data Availability

No new data were created or analyzed in this study. Data sharing is not applicable to this article.
